# Ovotesticular Differences of Sex Development: Surgery or Not Surgery? That Is the Question

**DOI:** 10.3389/fped.2017.00231

**Published:** 2017-10-31

**Authors:** Maria-Grazia Scarpa

**Affiliations:** ^1^Unity of Pediatric Surgery, Department of Surgery, IRCCS Materno Infantile Burlo Garofolo (IRCCS), Trieste, Italy

**Keywords:** ovotesticular DSD, fertility, genital ambiguity, treatment, surgery

The assignment of the sex of rearing for patients with precocious diagnosis of ovotesticular difference of sex development (OT-DSD) is a complex decision.

Ovotesticular disorder or difference of sex development (OT-DSD) is a very rare congenital anomaly characterized by the simultaneous presence of both testis and ovary in the same individual. It occurs between 3 and 10% of the total DSD (1). The most common karyotype is 46 XX (60%), followed by chromosomal mosaicism/chimerism (33%) and 46 XY karyotype (7%) (2). Gonadal tumors occur between 2.6 and 4.6% of OT-DSD, more frequently in 46, XY cases (3). Therefore, a rigorous follow-up is needed. About Müllerian remnants; the possibility of degeneration is exceptional, although some authors describe it (4). There is no evidence that prophylactic removal of asymptomatic Müllerian remnants is required. If possible, we suggest leaving them *in situ*: they can theoretically be useful in sex-reverse genitoplasty.

The timing of surgery for OT-DSD remains contentious, especially after the Consensus Statement on Management of Intersex Disorders held in Chicago in 2005. During this Meeting, international experts assembled to review this topic and to establish guidelines to help optimize and modernize the care given to patients with DSD. Gender assignment in newborns with OT-DSD represents a therapeutic challenge, the factors that influence gender assignment include:

– Diagnosis
– Genital appearance
– Surgical options
– Need for life long replacement therapy
– Potential for fertility
– Views of the family
– Circumstances relating to cultural practice (5).

According to recent International trends, it is better to postpone surgery and maintain an indeterminate gender until the patient can participate to the decision. Most children with OT-DSD present ambiguous genitalia as newborns or infants. Rarely, OT-DSD has been detected later in individuals with female or male normal phenotype.

During infancy, if a surgical operation is necessary, the parents can only give their consent. The question is: masculinizing or feminizing genitoplasty in OT-DSD is “a necessary operation” in pediatric age?

The goal of DSD therapy is the welfare of patients inside their families and in the society.

The recent increased acceptance of gender variance in society potentially facilitates an easier future for patients with DSD than anytime in modern history.

Nevertheless, a recent Japanese research (6) shows the preference of most families for early genital surgery in the case of OT-DSD. In Italy, families often want to have a defined sex of the child: they could not cope him or her to be stuck in the middle even if a specific counseling and a psychological support has been given. Most of the Italian parents, like Japanese ones, ask for early surgical solutions to ensure child well-being within the family, the school, and the society. This is in contrast with the recent Human Right Movements and the International trends. According to expert opinions, a precocious operation is not indicated. Patient’s consent to surgery would be preferable and a delayed surgery would be the best solution.

The psychologist plays an important role in supporting the specialists in the interaction with the family and helps parents to overcome the discomfort after diagnosis communication and after any medical or surgical intervention. His responsibility is to give regular consultancies and constant support both to families (accepting the gender or no gender assignment) and the patient in the critical phases of growth and personal and sexual development.

We consider families’ opinion about the sex of rearing an important issue, like cultural aspects and views. Society is changing but the sexual binary concept is not already overcome.

After specific psychological evaluations and after gender assignment by DSD team, if family asks for an early surgical correction of genital ambiguity, we respect its request, considering parents’ consent a crucial target.

We consider early surgery necessary in the following cases:

(1) 46 XY karyotype or for mosaicism containing Y material, male-like external genitalia, and androgen response to hCG test: in OT-DSD cases with male dominance, the negative feedback effect of ovarian steroids suppressing gonadotropins results in tubular atrophy, poor germ cell development, Leydig cell hyperplasia and sclerosis, and finally in infertility of testicular tissue (7)
(2) Female-like external and internal genitalia and negative hCG test even if karyotype contains Y material. In fact, when no functional chance in male sense exists, the risk of malignant degeneration can’t be neglected
(3) Presence of histologically confirmed streak gonads

Nowadays, the surgeon plays a difficult role: what does he do when families refuse a baby with genital ambiguity? Is it sufficient a surgical consent form with a clear explanation of both advantages and consequences of surgery versus no surgery?

A recent research article about OT-DSD treatment shows that even if ethicists and patient support groups advocated that the genital surgery should not be warranted until the patient was able to give the informed consent, the appearance of bisexual phenotype and continuous anxiety of parents call for the management. The authors conclude that it is rational and mandatory to initiate the evaluation at an early age (7).

We think that a multidisciplinary approach is essential: excellent medical, surgical, and psychological expertise is required in the treatment of OT-DSD. Good cosmetic result after surgical treatments and the respect of potential for fertility are mandatory.

In our opinion, the welfare of the patient in the family and in the society is an undeniable right.

To date, only few studies about gender dysphoria in this group of patients and no specific well-coded guidelines exist. Matsui et al. report their experience with eight OT-DSD children and reviewed 165 cases of OT-DSD from Japanese institutions. According to their 20-year experience in Japan, despite informing families of all treatment options available, they have often desired early gonadal surgery and genitoplasty. On an 8.2-year mean follow-up, none of their patients had gender identity disorder or gender dysphoria (6).

In a retrospective analysis of 64 OT-DSD cases in South Africa, the most common karyotype was 46,XX (88%), followed by 46,XY (8%), 46,XY/45,X (3%), and 46,XX/46,XY: the male gender was the predominant sex of rearing in two-thirds of the subjects and gender dysphoria was noted in eight patient (11%) at a median of 6.4 years; long follow-up revealed dysphoria in two cases and neuropsychiatric disorders in four cases (8).

We refer to the general principles of the Consensus Conference of Chicago (5) for proper management of these patients.

The psychologist has a key role in the multidisciplinary team: he supports the family who agrees to postpone surgery and highlights parents who are unable to accept a child with genital ambiguity.

Our future aims are:

– To collect new cases of OT-DSD and participate to multicenter studies
– To create a surgical consent form with a part reserved to the psychological interview.

## Author Contributions

The author wrote, reviewed the manuscript, and performed the literature search.

## Conflict of Interest Statement

The author declares that the research was conducted in the absence of any commercial or financial relationships that could be construed as a potential conflict of interest.
